# A Hierarchical Topology Control Algorithm for WSN, Considering Node Residual Energy and Lightening Cluster Head Burden Based on Affinity Propagation

**DOI:** 10.3390/s19132925

**Published:** 2019-07-02

**Authors:** Ling Song, Qidong Song, Jin Ye, Yan Chen

**Affiliations:** 1 School of Computer & Electronic Information, Guangxi University, Nanning 530004, China; 2 Guangxi Key Laboratory of Multimedia Communications and Network Technology, Nanning 530004, China

**Keywords:** WSN, topology control, double cluster heads, multi-hop, energy consumption, round

## Abstract

The low energy adaptive clustering hierarchy (LEACH) is the classical hierarchical topology control algorithm and still widely used today in wireless sensor networks. There are some problems in LEACH such us an unreasonable selection and uneven distribution of cluster heads, not considering the residual energy of nodes, the cluster head is overloaded and there is a high energy consumption of data transmission. In this paper, a topology control algorithm with double cluster heads and multi-hop based on affinity propagation clustering (APDC-M) was proposed. In APDC-M, firstly, a cluster head election algorithm with double choices based on the affinity propagation clustering algorithm (AP) and reference node strategy (APDC) was proposed. APDC is responsible to elect the fusion cluster head for node clustering. Secondly, a forwarding cluster head is elected within each cluster. The forwarding cluster head is responsible for the data transmission in order to reduce the energy consumption of a single cluster head. Lastly, the forwarding cluster heads complete the data transmission from a cluster to the base station by the shortest path inter-cluster multi-hop mode. The comparison simulation results show that APDC-M can make the cluster head election more reasonable and the cluster head distribution more uniform, and effectively reduce the energy consumption of the cluster head nodes when transmitting data, thus prolonging the network life.

## 1. Introduction

Wireless sensor network (WSN) is a large-scale network composed of a large number of sensor nodes in the form of self-organization. Although the deployment of WSN is convenient and the cost is low, there are some problems such as limited node energy and short network lifetime. The WSN topology control is a reasonable design method for node transmission power, network structure and other factors. Different from the traditional network topology control technology, the WSN topology control technology should reduce the workload of nodes as much as possible, control node energy consumption, dynamically update topology and simplify the routing mechanism between communication nodes. Thus, the purpose of reducing the energy consumption of the whole network and prolonging the life of the network is achieved. Topology control is one of the core problems in the field of WSN.

Using the appropriate network topology control algorithm to optimize the network structure can effectively save node energy consumption and thus prolong the service life of the network. So, the study on the network topology control algorithm is of great significance. Many scholars have proposed a series of WSN topology control algorithms to improve the performance of the network. According to the research direction, they can be divided into a power-based topology control algorithm and hierarchical topology control algorithm. This paper mainly studies the improvement of the hierarchical topology control algorithm. The low energy adaptive clustering hierarchy algorithm (LEACH) proposed by Heinzelman W. R. et al. in 2000 in [1] is the earliest and representative of the hierarchical topology control algorithm. LEACH is a multi-round topology control algorithm, it firstly introduces the concept of cluster structure instead of the previous flooding mode, which can effectively reduce the energy cost of the network. The LEACH elects cluster head nodes in each round, and then the cluster size and position are determined according to the cluster head node position. The concept of alternating cluster head avoids the early death of some cluster heads due to excessive energy consumption; the calculation method also introduces data fusion technology, the cluster head is responsible for data fusion and forwarding, and ordinary nodes enter dormancy state in a non-working time slot to effectively reduce the energy consumption of network nodes. Clustering greatly reduces the transmission distance of the nodes in the network, thus saving the energy consumption of the nodes and increasing the effective working time of the network.

Some scholars also studied on hierarchical topology control after LEACH was proposed, such as Xu Yi presents a clustering algorithm (GAF) based on the actual geographical location in [2], GAF abstracts graphics of monitored areas and splits them, then allocated data on the splitted graphics based on the node geolocation. The algorithm needs the node to have the geographic information recognition system, so the function of the equipment is required; in [3], an algorithm with cluster head tree association structure, which can quickly cluster in the networks with high node density (TopDisc), is proposed. The algorithm has the problem of low flexibility and is not suitable for application in the networks with great changes of environment; The HEED algorithm proposed in [4] sets the primary and secondary parameters to complete clustering, which can solve the problem of uneven clustering to a certain extent, but the algorithm is easy to generate isolated nodes. These are the small tributaries of research on hierarchical topology control.

Due to the good performances of LEACH, it has been the basis of many hierarchical network topology control algorithms since it was presented. However, the LEACH algorithm has the following main problems: (1) The distribution of cluster head nodes is uneven. LEACH selects cluster heads randomly, there will be an uneven distribution of nodes, which will lead to large differences in the scale of each cluster in the network, resulting in the early death of some cluster head nodes due to excessive burden; (2) the low energy node may be elected as a cluster head. Since LEACH does not consider the residual energy of the node in the election of the cluster head, the low energy node may act as the cluster head, which will lead to the uneven energy consumption of the nodes in the network, resulting in the premature death of some nodes, which will affect the overall network performance.

It is precisely because the classical LEACH has many advantages and needs to be improved at the same time that it becomes the research basis of many later studies and scholars put forward some improvements to it. However, these improved algorithms have the following problems: Some do not consider the residual energy of the node when selecting the cluster head; some only consider the selection of high energy nodes as cluster head nodes without considering the influence of cluster head node position; some can not produce the ideal results after the death of network nodes, and there is the uneven energy consumption of cluster head nodes in different positions. See Section 2 for details about these.

Like most scholars, our study also focuses on the improvement of the classical LEACH and tries to do better than the same kind of improvements. A hierarchical topology control algorithm with double cluster heads and multi-hop based on affinity propagation clustering (APDC-M) is proposed in this paper. The main contributions of this paper are: (1) Because the cluster head election of WSN is similar to the cluster center election of the clustering algorithm, this paper proposes a WSN topology control algorithm based on affinity propagation clustering (AP) to solve some problems existing in classical LEACH and some improved algorithm of LEACH, in which, the residual energy of the node is introduced. During the process, an election strategy based on the reference node is proposed and selectively alternative work in order to reduce the amount of computation of the cluster head election. This strategy can make the distribution of the cluster head more reasonable, and make the clustering more evenly; (2) with considering the residual energy of the nodes, the algorithm elects two cluster heads for each cluster from the point of view of reducing and balancing the communication energy consumption of the nodes. One cluster head is responsible for the data fusion within the cluster, and the other cluster head transmits the fused data to the base station by means of multi-hop between clusters. These above strategies of APDC-M can achieve the goal of ultimately prolonging the overall life of the network.

The rest of the paper is organized as follows: The Section 2 is the related work about LEACH; in the Section 3, the LEACH and AP algorithms are introduced; the Section 4 describes the design of the APDC-M algorithm in detail; the Section 5 gives the experimental results and analysis based on the NS2 simulation platform; and the Section 6 is the summary of this paper.

## 2. Related Work

As this paper is based on the classical LEACH for improvement, the following mainly introduces the related research status around LEACH.

The LEACH algorithm proposed in [1] is the earliest hierarchical topology control algorithm with the concept of clustering, and is the foundation of most self-organizing clustering algorithms. The method of constructing clusters by LEACH algorithm can reduce the energy consumption of the whole network. However, in the process of cluster head election, LEACH has some problems such as not considering the residual energy of the node, uneven distribution of cluster head nodes and unbalanced energy consumption of each node. In [5], an improved algorithm LEACH-C is proposed, in which all nodes send their position information and residual energy value to the base station in each round. After receiving this information, the base station first calculates the average energy of all nodes, and takes the node whose energy is not less than the average energy as the candidate node. This method can reduce the energy consumed by communication in the election of cluster heads, so that more residual energy can be used to transmit data, and the nodes with higher residual energy can be selected as cluster heads, but the algorithm still has the problem of unreasonable location of cluster head nodes. The EBCRP algorithm proposed in [6] optimizes the election mode of the LEACH cluster head, so that the cluster head nodes can be evenly distributed in the network after election, so as to balance the energy consumption of the network as a whole. However, the high complexity of the algorithm requires high communication and computing capability of nodes. The energy optimized LEACH algorithm (LEACH-EC) proposed in [7] and the low energy consumption LEACH algorithm (O-LEACH) proposed in [8] take into account the influence of the residual energy of each node in cluster head election. It can ensure that the cluster head nodes have high residual energy, but the distribution of the election results is still unreasonable. In [9], a LEACH-MAC algorithm is proposed to control the number of cluster heads, which can stabilize the number of cluster heads in an ideal range and effectively reduce the randomness of the LEACH algorithm, but the distribution of cluster head nodes is not ideal. In [10], an internal equalization algorithm, IBLEACH algorithm is proposed. The algorithm extends the network life by balancing the energy consumption of each node, but some nodes will produce ineffective energy consumption. In [11], an improved LEACH algorithm (LEACH-KM-GA) based on K-means clustering algorithm is proposed. This algorithm introduces the idea of K-means algorithm in the process of cluster head election, which can obtain more reasonable election results. However, the dependence on the election of the initial cluster center is strong. In [12], an improved LEACH algorithm based on energy consumption gradient (EDROPL) is proposed. This algorithm can reduce and balance the energy consumption among nodes by dividing the region of the network and completing the cluster head election through the evaluation function. However, the algorithm needs the base station node to be in the center of the network as far as possible. In [13], an improved LEACH algorithm that partitions the network into sectors (pLEACH) is proposed to balance the energy consumption of the network by electing the nodes with higher energy in each partition as cluster head nodes. However, the problem of fast energy consumption of cluster heads in more nodes will occur. The AP-LEACH algorithm proposed in [14] is based on AP clustering during the cluster head election, and can elect cluster head nodes with reasonable distribution according to different network environments. There are also problems in computing and communication traffic in the election process.

In summary, at present, the main research directions of hierarchical topology control algorithms in WSN can be divided into the following two aspects:

1. Optimize the election method of the cluster head. As the core work of hierarchical topology control algorithm, the election and distribution of cluster head nodes directly affect the quality of the whole algorithm and become the important indexes to measure the performance of the algorithm. Unreasonable cluster head election results will greatly increase the energy consumption burden of cluster head nodes, lead to premature death of nodes and shorten the lifetime of the whole sensor network. Therefore, the research of the cluster head election is one of the hot research directions of the hierarchical topology control algorithm.
2. Reduce data energy consumption of transmission. Nodes of WSN will consume a lot of energy when transmitting data, different transmission paths and transmission modes will show obvious differences in energy consumption. Therefore, how to construct a more reasonable transmission path is a hot topic in the current topology control algorithms.

In this paper, considering the problems existing in the above-mentioned researches, the following improvements were made: A double cluster-head and multi-hop topology control algorithm (APDC-M) based on AP clustering was proposed. Firstly, the clustering strategy based on AP and reference node was used to elect the fusion cluster head, which is responsible for data fusion in a cluster; secondly, to lighten the burden of the cluster head, the forwarding cluster head was elected within each cluster to take charge of the external data transmission; thirdly, when transmitting data from cluster head to base station, the multi-hop method among cluster heads was used instead of the single-hop transmission in the LEACH algorithm, so as to minimize the energy consumption of transmission.

According to the above analysis, a brief comparison among the recent works are as Table 1.

All of the algorithms in Table 1 improve LEACH. Where, EBCRP, EDROPL, pLEACH and APDC-M perform well in the rationality of cluster head distribution and the balance of cluster head energy consumption. EBCRP modifies the random formula of cluster head election in LEACH, and introduces the idea of an empirical factor to prolong the network life. However, according to different network environments, the empirical factor of the algorithm needs to be re-simulated before it can be determined. Compared with EBCRP, APDC-M can better adapt to different network environments and has higher flexibility. EBCRP does not consider the residual energy of the node, so the rationality of cluster head election will be lower than that of APDC-M. EDROPL is suitable for the scene where the base station is located in the center of the network, and when the distribution of nodes is uneven, the energy consumption gap of the network nodes is large. However, APDC-M has no high requirements for the location of base station and the distribution of network nodes. Both of EBCRP and EDROPL do not consider the residual energy of the node, so the rationality of cluster head election is inferior to that of APDC-M. Compared with pLEACH, APDC-M effectively controls the gap between the number of nodes in each cluster and reduces the problem of uneven energy consumption of cluster heads caused by the large number of nodes in the cluster. Although EBCRP, EDROPL and pLEACH adopt the multi-hop transmission mode from cluster head to base station, each cluster has only one cluster head, which is responsible for both data fusion and data transmission. The burden of the head is heavy, this will affect the overall survival time of the network. The AP-LEACH proposed in [14] is also an improved LEACH algorithm based on AP clustering, which will be compared in the Section 5. Generally, the APDC-M algorithm in this paper has advantages in all the above aspects.

## 3. LEACH and AP

### 3.1. LEACH Algorithm

Low energy adaptive clustering hierarchy algorithm (LEACH) establishes multiple hierarchies through cluster head election and clustering strategy, and reduces communication energy consumption through intra-cluster forwarding. The algorithm works periodically in the network, each work round is divided into two phases: The negotiation phase and stable phase.

In the negotiation phase, the cluster is constructed, the cluster head node is elected and the cluster is formed according to the position of cluster head, this phase is also responsible for the initialization of the algorithm. If a node is elected as a cluster head, it sends its own ID and other information to other nodes by broadcast. After receiving this information, the other nodes that are not elected as the cluster head will determine their own cluster head according to the signal strength, and a message is sent to the cluster head for joining the cluster. The cluster head forms a list of intra-cluster node information, and allocates communication slots for each intra-cluster node so as to avoid intra-cluster communication conflicts. The main work in the stable phase is data transmission. The nodes in the cluster transmit the collected data to the cluster head in the time slot allocated by the cluster head, and then the cluster head sends data to the base station after processing. When all nodes finish sending data, this round of work ends and a new round begins.

By introducing the concept of the cluster structure to replace the previous flooding mode, the LEACH algorithm effectively reduces the energy overhead of the network; and the concept of rotating cluster head proposed in the algorithm avoids the premature death of some cluster head nodes due to excessive energy consumption. The algorithm also introduces the data fusion technology, the cluster head node is responsible for data fusion and forwarding, and the common node enters sleep state in the non-working time slot, which effectively reduces the energy consumption of the network node. However, the LEACH algorithm also has the following problems:

1. The randomness of cluster head election is too strong. LEACH algorithm elects the cluster head randomly, which leads to the uneven distribution of nodes, resulting in a large difference in the size of each cluster in the network. It makes some cluster head nodes die early because of overburden.
2. The low energy node is elected as cluster head. Leach algorithm does not consider the residual energy of cluster head, and the lower energy node may act as cluster head, which results in the imbalance of energy consumption and premature death of some nodes in the network, these will affect overall network performance.

### 3.2. AP Clustering Algorithm

AP is a new unsupervised clustering algorithm, it does not need to pre-set the location and number of clustering centers, but through the information iteration of data points to determine the appropriate clustering centers. The sum of similarity between all data points and the nearest cluster center is maximized. This algorithm can be used for fast convergence clustering under less constrained conditions [15]. Therefore, AP is used in the clustering of self-organizing networks. The process of the algorithm is:

Step 1. The similarity s(i, j) between node i and node j is calculated and stored in a similarity matrix S. The size of the similarity is expressed as a negative number of Euclidean distance, as shown in formula (1), where d_i,j_ is the Euclidean distance between i and j. It means that the greater the distance between i and j, the smaller the similarity.

(1) $$s(i,j)=-d_{i,j}.$$

Step 2. Initializing the attractivity matrix R and attribution matrix A, and setting the values of attractivity r(i, j) and attribution degree a(i, j) between node i and node j to be 0.

Step 3. Iteratively updates r(i, j), a(i, j), the formulas for updating are shown in Formulas (2)–(5), where, λ is the attenuation factor introduced while iterative updating, its value is between 0.5 and 1, r (i, j), a (i, j) are the calculated values of this round, r′(i, j), a′ (i, j) are the calculated values of last round.

(2) $$r(i,j)=\{\begin{array}{ll} s(i,j)-\underset{j^{\prime }\neq j}{\max}\left[a(i,j^{\prime })+s(i,j^{\prime })\right] & ,i\neq j\\ s(i,j)-\underset{j^{\prime }\neq j}{\max}[s(i,j^{\prime })] & ,i=j \end{array},$$

(3) $$a(i,j)=\{\begin{array}{ll} \min\left\{0,r(j,j)+\sum_{j^{\prime }\neq j,i}\max\left[0,r(j^{\prime },j)\right]\right\} & ,i\neq j\\ \sum_{i^{\prime }\neq j}\max\left[0,r(i^{\prime },j)\right] & ,i=j \end{array},$$

(4) $$r(i,j)=\lambda *r^{\prime }(i,j)+(1-\lambda )*r(i,j),$$

(5) $$a(i,j)=\lambda *a^{\prime }(i,j)+(1-\lambda )*a(i,j).$$

Step 4. Each node sums the attraction and attribution values and elects the largest one as its clustering center. If the clustering result is in accordance with the expected value or the number of iterations reaches the preset value, the algorithm ends.

## 4. APDC-M Algorithm

### 4.1. Basic Idea

Due to the strong commonness between cluster head election and finding clustering center of the AP algorithm, a cluster head election strategy based on AP was proposed, which transforms cluster head election process into searching cluster center. The energy consumption of receiving and transmitting data between nodes is taken as a measurement of similarity, and the residual energy of nodes is used as an influence factor to calculate the attraction between nodes. In the cluster head election, all the nodes are regarded as potential cluster head nodes, and the nodes are eliminated from each other by cyclic iterative calculation. Finally, for each cluster, the node that is most suitable for the cluster head is elected as the fusion cluster head and complete clustering. Considering the burden of a single cluster head, we elected another node in each cluster to transfer the data to the base station, then constructed the multi-hop path among the transfer cluster heads of clusters based on the shortest path algorithm.

### 4.2. Election of Double Cluster Heads

#### 4.2.1. Election of Fusion Cluster Head (APDC)

In order to solve the problems in the election of cluster head of LEACH algorithm, we presented an adaptive double-election hierarchical topology control algorithm APDC based on AP clustering. Since the election strategy based on AP clustering has the defect of too much computation, an election strategy based on the reference point was added to make up for this defect. The two-policy alternation was used to complete the election of cluster head. Firstly, the election strategy based on AP clustering was used to complete the first round of cluster head election, and the election results were recorded as reference nodes. Each round was completed by alternately using these two election strategies according to the validity of the reference nodes. The two election strategies of APDC make up for each other’s shortcomings in the process of rotation, so that the algorithm can adapt to the dynamic changes of sensor networks, such as the changes of energy consumption and the changes of node distribution due to node death, and obtain more reasonable election results of cluster heads.

1. Cluster head election based on AP clustering.

The specific workflow for this strategy is as follows. The meaning of the parameters in the following formulas of the workflow steps is shown in Table 2.

Step 1. The similarity between nodes was calculated, and the initial value of the attraction degree and attribution degree between nodes was set to 0.

In calculating the similarity between nodes, according to the special requirements of WSN to cluster head nodes, taking the energy consumption model of WSN as a reference, the energy consumption of data transmission between nodes was taken as a measurement of the similarity between nodes. The specific formula is shown in (6), where, d_i,j_ is the distance from node i to node j, and d_i,bs_ is the distance from node i to base station node.

(6) $$\{\begin{array}{l} s(i,j)=-\left(E_{elec}+\varepsilon_{fs}*d_{i,j}{}^{2}\right),i\neq j,d_{i,j}<d_{0}\\ s(i,j)=-\left(E_{elec}+\varepsilon_{mp}*d_{i,j}{}^{4}\right),i\neq j,d_{i,j}>d_{0}\\ s(i,j)=-\left(E_{elec}+\varepsilon_{mp}*d_{i,bs}{}^{4}\right),i=j \end{array},$$

$$\mathrm{Where},d_{0}=\sqrt{\varepsilon_{fs}/\varepsilon_{mp}}.$$

Step 2. Calculate the attraction between nodes.

When calculating the degree of attraction between nodes, the residual energy E_rem(i)_ of the current node was introduced as the influence factor, as shown in the formula (7), where, E_init_ represents the initial energy of the node. The smaller the remaining energy of the nodes, the lower the attraction of the nodes to other nodes and the lower the possibility of becoming the cluster head nodes, so as to avoid the nodes with lower energy becoming cluster heads.

(7) $$r(i,j)=\{\begin{array}{ll} \left(1-\frac{E_{rem}(i)}{E_{init}}\right)*s(i,j)-\underset{j\neq j^{\prime }}{\max}\left[a(i,j^{\prime })+s(i,j^{\prime })\right] & ,i\neq j\\ \left(1-\frac{E_{rem}(i)}{E_{init}}\right)*s(i,j)-\underset{j\neq j^{\prime }}{\max}[s(i,j^{\prime })] & ,i=j \end{array}.$$

Step 3. Formula (3) was used to calculate the value of attribution degree between nodes.

Step 4. The cluster center of each node was determined.

The clustering center (cluster head) of the node was determined by summation of the attraction degree and the attribution degree of the node, as shown in formula (8). On the premise of maximum sum, if i equals to j, i (or j) is the cluster head of itself, otherwise j is the cluster head of i. By judging the clustering center of each node, the cluster head to which each node belongs was obtained.

(8) sum=max(a(i,j)+r(i,j))

Step 5. The current number of iterations plus 1. If the cluster centers (that is, cluster heads) remained unchanged after multiple iterations, or the total number of iterations reached the set maximum, (these two values should be determined in terms of the specific experiment scene of WSN, in this paper, they were set to be 20 and 200, respectively), the ID information of cluster head was broadcasted by the base station, and the iterative process of the election algorithm based on AP clustering was ended. Otherwise, jump back to Step 2 to continue the iteration.

2. Cluster head election based on the reference node.

In the reference-node-based election strategy, the set of reference nodes a(i) was the final collection of cluster head nodes obtained by the last round election strategies based on AP clustering, the workflow is as follows:

Step 1. The cluster head was elected based on the reference node.

The election strategy based on reference node calculated the differences between other nodes and the reference node to conduct cluster head election. The formula of similarity was as (9), the reference node i calculates the difference G(i,j) between each non-reference node j and it respectively, and elects the node j whose G(i,j) is the smallest as the cluster head of this round.

(9) $$G(i,j)=\left(d_{i,j}+d_{i,j}*d_{j,bs}\right)*\frac{1}{E_{rem}}.$$

This election strategy takes into account the following influencing factors: Residual energy E_rem_ of the non-reference nodes, the distance d_i,j_ from the non-reference node j to the reference node i and the distance from the non-reference node j to the base station d_j,bs_, which makes the elected cluster head nodes evenly distributed, the cluster heads have higher residual energy and they are relatively close to the base station.

Step 2. If the election result was valid, the election work was completed; otherwise, it alternated to the election strategy based on AP clustering and re-elected the cluster head.

After node j was elected as the cluster head of this round, the validity of the cluster head was determined by the judgment function, which is shown in formula (10), where E_rem_ represents the current residual energy of node j, E_aver_ is the average residual energy of all living nodes in the network, d_i,j_ is the distance between the node j and the reference node i and d_aver_ is the average distance between the child nodes and the reference node i when i is the cluster head of last round. The elected cluster head j is valid only if the value of p(j) equals to 1. If all elected cluster heads are valid, the clustering is successful; otherwise, alternate to the election strategy based on AP clustering to re-cluster and update the reference nodes.

(10) $$p(j)=\{\begin{array}{l} 0,E_{rem}<E_{aver}\\ 0,d_{i,j}>d_{aver}\\ 1,\mathrm{other} \end{array}.$$

After the fusion cluster head election was completed, the cluster heads broadcasted to the other codes. According to the received signal intensity, a non-cluster head node selects the cluster head node with the strongest signal as its cluster head.

#### 4.2.2. Election of Forwarding Cluster Heads

In order to reduce the burden of the fusion cluster head, another forwarding cluster head was elected to transmit the data to the base station. The fitness that each common node in the cluster can be elected as the forwarding cluster head is based on the energy consumption of the node sending to the base station, and the residual energy of the node is taken into account to avoid the node with too low energy to be elected. The derivation process of the fitness formula is as follows:

1. Assuming that node i is a node in the cluster, because the free space model is used for intra-cluster communication, the calculation of the energy consumption E_fus_ that a fusion cluster head transmits k bits data to node i is shown in formula (11), e.g., [1].
  (11) $$E_{fus}=k*E_{elec}+k*\varepsilon_{fs}*d_{i,f}{}^{2}.$$
2. Assuming that the node i has been elected as the forwarding cluster head, using the multipath fading model when forwarding data to the base station, the calculation of energy consumption E_send_ is shown in formula (12), e.g., [1].
  (12) $$E_{send}=k*E_{elec}+k*\varepsilon_{mp}*d_{i,bs}{}^{4}.$$
3. Considering the influence of the above two kinds of energy consumption and the residual energy of the node, the fitness formula of the node i to be forwarding cluster head is deduced as shown in (13). The smaller the C value is, the more suitable the node is to be elected as forwarding cluster head.
  (13) $$C(i)=\frac{E_{init}}{E_{rem}(i)}*\left(\varepsilon_{fs}*d_{i,f}{}^{2}+\varepsilon_{mp}*d_{i,bs}{}^{4}\right).$$

### 4.3. Multi-Hop Transmission Based on the Shortest Path

When the forwarding cluster head of each cluster transmits data to the base station, the path election from the cluster head to the base station directly affects its communication energy consumption, so it is necessary to consider how to make the forwarding cluster head forward the data to the base station through the most reasonable path. In this paper, the multi-hop transmission mode among cluster heads is used to replace the single-hop mode of LEACH.

**Figure 1:**
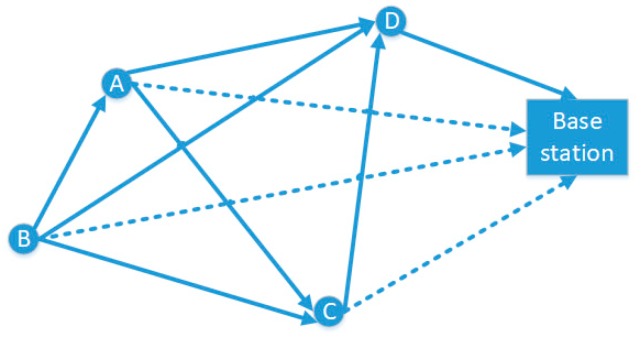
Network diagram of the base station and forwarding cluster heads.

Assuming that the elected forwarding cluster heads are A, B, C and D, the whole network can be simplified as a graph of the base station and the forwarding cluster heads, as shown in Figure 1. The solid lines are the forwarding path between the cluster heads, the path direction is that the cluster head, which is far from the base station point to the cluster head, which is close to the base station; and the dotted line is the single-hop forwarding path between each cluster head and the base station.

According to the multi-path fading energy consumption model and considering the residual energy of the node to which the forwarding path is directed, the cost of the forwarding path was calculated. As shown in formula (14), P_cost_(i,j) is the path cost of node i to node j, and d_i,j_ is the distance between i and j.

(14) $$P_{\cos t}(i,j)=\frac{E_{init}}{E_{rem}(j)}*\left(k*E_{elec}+k*\varepsilon_{mp}d_{i,j}{}^{4}\right).$$

Since the influencing factors of path weight are only related to the residual energy of the destination node and the distance between the two nodes, the formula (14) was simplified to formula (15), which was used to calculate the path cost between the two nodes.

(15) $$P_{\cos t}(i,j)=\frac{E_{elec}+\varepsilon_{mp}d_{i,j}{}^{4}}{E_{rem}(j)}.$$

When calculating the path cost between the cluster head and the base station, because the energy of the base station is infinite in theory, only the distance between the cluster head and the base station was considered as the influencing factor, and the calculation of P_cost_(i,bs) is shown in (16).

(16) $$P_{\cos t}(i,bs)=\varepsilon_{mp}d_{i,bs}{}^{4}.$$

**Figure 2:**
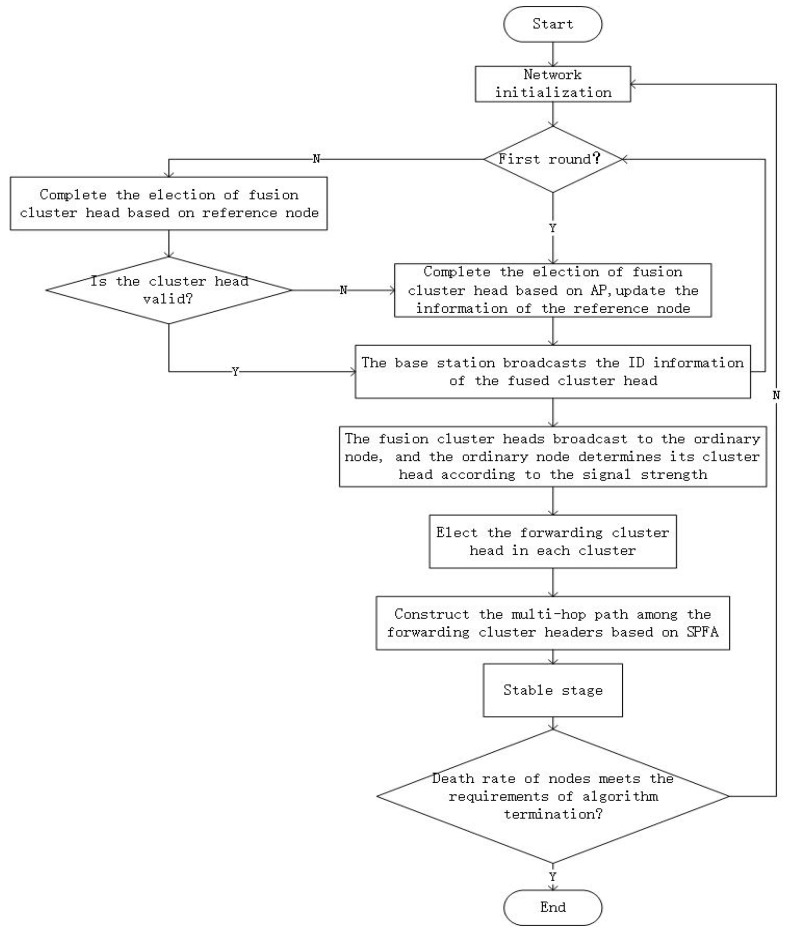
The overall workflow of APDC-M.

The parameters of the above formula are defined in Table 1. After calculating the cost of all forwarding paths, the base station was regarded as the source point and all the path arrows in Figure 2 were reversed, we obtained a directed graph with weight. In this case, the problem of finding the best multi-hop path from the cluster head to base station could be abstracted to find the shortest path from the source point to the other points. Since the distribution of the forwarding cluster head nodes was relatively sparse and the whole network required high computational efficiency, the APDC-M algorithm in this paper was based on the efficient shortest path faster algorithm (SPFA) in [16] to complete the path planning from the source point to other nodes. From the analysis of references [17,18,19], compared with Dijkstra algorithm, Ford algorithm and Bellman-Ford algorithm, the SPFA algorithm had lower time complexity.

The main idea of the SPFA algorithm is to save the relaxed nodes with first-in first-out queue and iterate continuously to find the shortest path. Compared with the Dijkstra algorithm, Bellman-Ford algorithm and other algorithms for finding the shortest path, the SPFA algorithm had lower time complexity.

The specific implementation methods of SPFA algorithm are as follows:

Step 1. Establish a queue and table to save node information and the shortest path information from the source point to other nodes respectively.

Step 2. Initialize the queue and the table, save the source point in the queue, all the values in the table are maximum, and the path length from each point to itself is 0.

Step 3. Perform a relaxation operation, update the shortest path information for all points in the table with the point out of the queue. If the updating is successfully updated and the updated point is not in the queue, the point is lined up.

Step 4. Repeat step 3 until the queue is empty, when the information in the table is the shortest path information required.

### 4.4. The Overall Process of APDC-M

In summary, the overall process of one round of APDC-M consists of three main strategies as follows:

Step 1. The stage of fusion cluster head election: The election strategy based on AP clustering or reference node strategy is used to complete the fusion cluster head election, and the common node selects the strong signal for the cluster head according to the broadcast signal strength of the cluster head node.

Step 2. The stage of forwarding cluster head election: Each node in the cluster calculates the forwarding cost and sends the cost information to the fusion cluster head. According to the cost information, the cluster head selects the forwarding cluster head and notifies the selected node. The forwarding cluster head broadcasts its elected information in the cluster, and transmits its energy and position information to the base station.

Step 3. The stage of multi-hop path construction: The base station uses the inter-cluster multi-hop strategy based on SPFA to determine the next-hop of each forwarding node, and the information is broadcast to each transferred cluster head.

The overall flow chart of APDC-M is shown in Figure 2.

### 4.5. Time Complexity of APDC-M

The APDC-M algorithm works on a round in WSN, so we analyzed the time complexity on the basis of one round, and we considered the time overhead of the looping iterative clustering. The time complexity of one round of the APDC-M algorithm was analyzed as follows.

#### 4.5.1. The Time Complexity of the Fusion Cluster Head Election

For the election strategy of the fusion cluster head, it was composed of two substrategies, and the two substrategies were used selectively in each round. The time complexity of the election strategy based on AP clustering was O(n2logn), the time complexity of the election strategy based on reference nodes was O(n), where, n is the number of network nodes. Combining the time complexity of the two substrategies, the time complexity of this overall strategy was O(n2logn).

It can be seen that the election strategy based on AP clustering can obtain more reasonable results, but the time cost is large. The election strategy based on reference nodes can obtain the results quickly, but with the change of network state, the reference nodes need to be updated from time to time to maintain the reasonableness of the election results. The two strategies complement each other, which makes the time complexity of APDC reasonable while obtaining the ideal cluster head election results.

#### 4.5.2. The Time Complexity of the Forwarding Cluster Head Election

The cluster with the largest number of nodes in the cluster was considered as the object, assuming that the number of nodes is n, the time complexity of the forwarding cluster head election strategy was O(n).

#### 4.5.3. The Time Complexity of SPFA

Assuming that the number of forwarding cluster head nodes was m, the time complexity was O (vm) when the APDC-M algorithm constructs the multi-hop paths among clusters based on SPFA, where v represents the average number of times all the cluster head nodes enter the queue. The algorithm uses lower time cost to improve the overload of cluster head nodes.

#### 4.5.4. The Time Complexity of the Overall Algorithm

APDC-M is an algorithm that consists of the above three subalgorithms: Fusion cluster head election, forwarding cluster head election and multi-hop path construction. Based on the fusion cluster head election with the highest time complexity, the overall time complexity of the APDC-M algorithm could be analyzed as O(n2logn).

## 5. Simulation Experiment and Result Analysis

In this section, the APDC-M algorithm was evaluated from two aspects: The number of network survival nodes and the network energy consumption, and the network simulator version 2(NS2) was used in the simulation experiments. NS2 is a free software simulation platform with open source code for network technology, using C and Otcl as the development language. Each of the following experiments was carried out 30 times, and the results were taken on average.

### 5.1. Experimental Scenarios

#### 5.1.1. Experimental Parameters

The main parameters of the experimental scenario are shown in Table 3.

#### 5.1.2. Network Model Setting

- All nodes in the network had the same initial energy and each node had a unique ID;
- After deployment, all nodes and base station in the network had fixed positions and did not move;
- The nodes in the network could adjust the transmission power according to the demand when carrying on the data communication;
- The base station node was outside the distribution area of the sensor nodes, its position was fixed and the energy was not limited.

#### 5.1.3. Initial Distribution of Network Nodes

**Figure 3:**
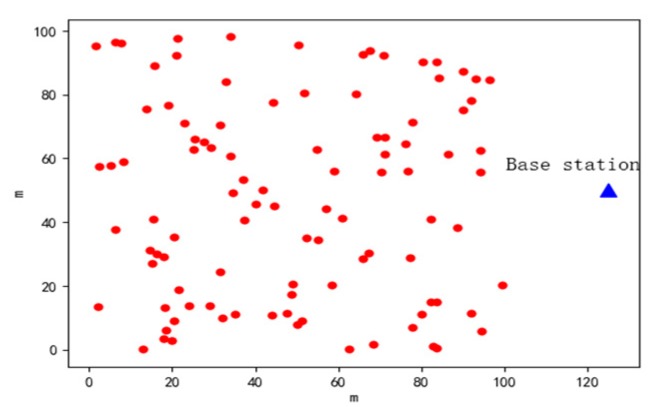
Network nodes initial distribution map.

The initial distribution map of the network nodes in this experiment is shown in Figure 3.

### 5.2. Network Energy Consumption Model

In this experiment, only the energy consumption of node receiving and transmitting data was considered in the energy consumption calculation, and the classical wireless communication energy consumption model was adopted. In the model, the energy consumption required by the node to send k bits data to the node with distance d is shown in formula (17), e.g., [1].

(17) $$\{\begin{array}{l} E_{tx}=k*E_{elec}+k*\varepsilon_{fs}*d^{2}\;,d<d_{0}\\ E_{tx}=k*E_{elec}+k*\varepsilon_{mp}*d^{4},d\geq d_{0} \end{array},$$

$$\mathrm{Where},d_{0}=\sqrt{\varepsilon_{fs}/\varepsilon_{mp}}.$$

The energy consumption of the receiving node to receive the data is shown in formula (18):

(18) $$E_{rx}=k*E_{elec}.$$

The meaning of the parameters in the above formula is shown in Table 4:

### 5.3. Simulation Experiment and Analysis

#### 5.3.1. Analysis of Cluster Head Node Distribution

In this experiment, the cluster-head distribution results of the LEACH algorithm were taken as the compared object. The main purpose was to evaluate the clustering results of APDC algorithm and analyze whether it could reach the expected target. Table 5 shows the comparison of the average values of clustering results of APDC and LEACH over the whole network life cycle, and no dead nodes existed in the network during statistics. It can be seen that compared with the LEACH algorithm, the difference of cluster size formed by the APDC algorithm was smaller, compared with LEACH algorithm, APDC could elect cluster head nodes with more reasonable distribution position, thus forming clusters of similar size and avoiding the overload of some cluster head nodes. The energy consumption of each cluster head node was balanced, and the communication cost between intra-cluster nodes and cluster heads was smaller.

**Figure 4:**
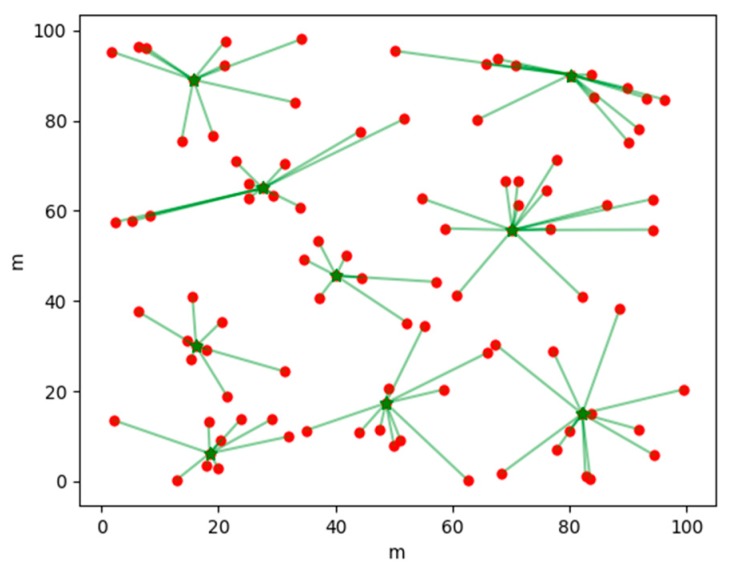
Cluster head distribution based on AP clustering strategy.

**Figure 5:**
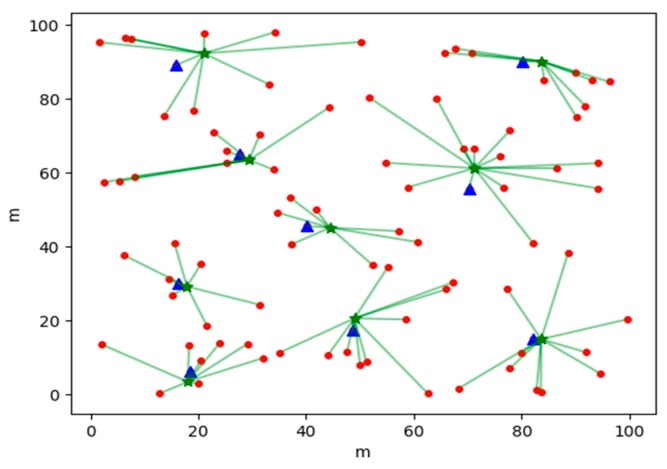
Cluster head distribution based on reference node strategy.

**Figure 6:**
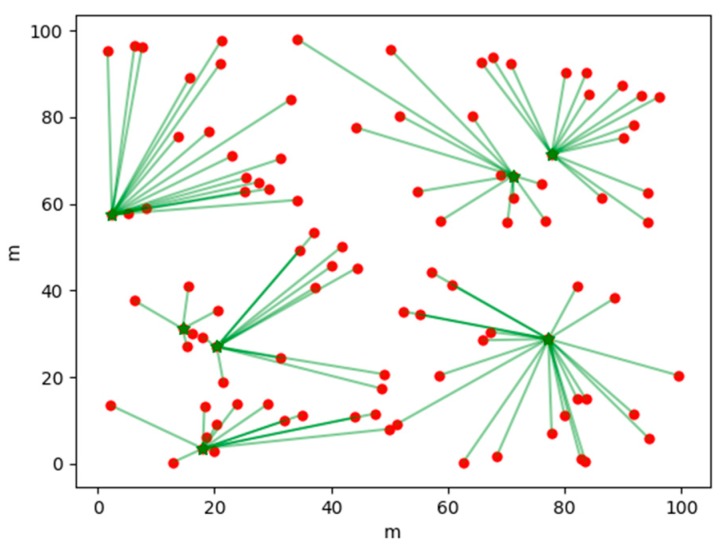
LEACH algorithm cluster head distribution.

Figure 4 shows the clustering results generated by a certain round of election strategies based on AP clustering in the APDC algorithm; Figure 5 is the clustering result generated by a certain round of election strategy of based on a reference node in APDC algorithm; and Figure 6 is a result of clustering of a certain round by the LEACH algorithm. The dots in the figures are common nodes, the triangles are the effective reference nodes of the current round, and the asterisk is a cluster head node after the clustering, the inner node of the cluster is connected with the cluster head by a solid line.

As shown in Figure 4, the APDC uses the election strategy based on AP clustering to elect the suitable cluster head nodes according to the distribution of nodes, and the size of the clusters is also more balanced. The reference node in Figure 5 is the cluster head node elected by clustering based on AP clustering election strategy in the last round, which can effectively reduce clustering time and energy consumption and produce more reasonable results. In Figure 6, because the distribution of nodes is not taken into account, the large randomness of LEACH algorithm leads to the uneven distribution of cluster head nodes, and the size of each cluster is also quite different.

#### 5.3.2. Analysis of the Survival of the Nodes

Assuming that the initial nodes had 100% energy, the whole time from the beginning of the algorithm to the total death of the nodes in the network due to energy depletion was the network lifetime referred to in this paper. The number of rounds in our simulation experiment reflected the network lifetime.

In this experiment, the survival of sensor nodes in the whole life cycle of WSN using the APDC-M algorithm was analyzed, the purpose of which was to evaluate whether APDC-M could achieve the expected goal in prolonging the effective life of the network and balancing the energy of the nodes. The experimental results are shown in Figure 6, where AP-LEACH was the algorithm in [14].

**Figure 7:**
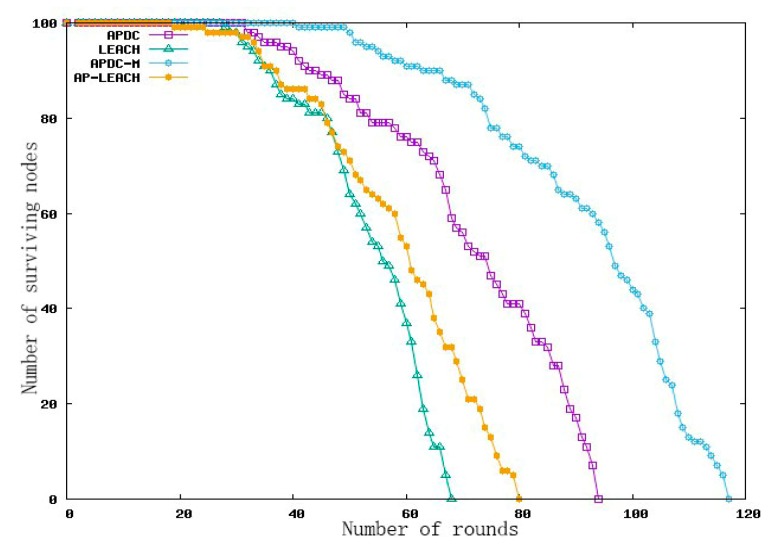
Comparison of node survival.

As can be seen from Figure 7, the LEACH algorithm had node death in 28 rounds, all nodes died in 68 rounds and the number of surviving nodes in the whole network was less than 50% after 50 rounds, and the overall performance of the network was poor. AP-LEACH had node death in 19 rounds and all nodes died in 80 rounds. Compared with LEACH, the whole network life cycle of AP-LEACH was extended by 12 rounds, but the node death time was 9 rounds ahead of LEACH. The reason for the early death of the node in AP-LEACH was that the residual energy of the node was not taken into account in the cluster head election, as a result, some nodes with more reasonable positions acted as cluster head nodes many times and died earlier, but compared with LEACH, because the distribution of cluster head nodes was more reasonable, the death speed of the whole network nodes was slow, and the network performance was better.

The APDC algorithm had dead nodes in 32 rounds and all nodes died in 94 rounds. Compared with AP-LEACH, the whole life cycle of AP-LEACH was extended by 14 rounds, and the time of node death was delayed by 13 rounds. The reason was that the two strategies used both took the residual energy of the node as the influencing factor, which avoided the overuse of the node with low energy, thus, greatly slowing down the death speed of the node.

Compared with APDC, the whole network life cycle of APDC-M algorithm was prolonged by 23 rounds, and the time of node death was delayed by 9 rounds. These data show that the double cluster heads and multi-hop strategies used in APDC-M could effectively balance the energy consumption among cluster head nodes, reduce the node death speed and avoid the problem of early large area death of the nodes far from the base station. The performance of the whole sensor network was improved.

#### 5.3.3. Analysis of Energy Consumption

**Figure 8:**
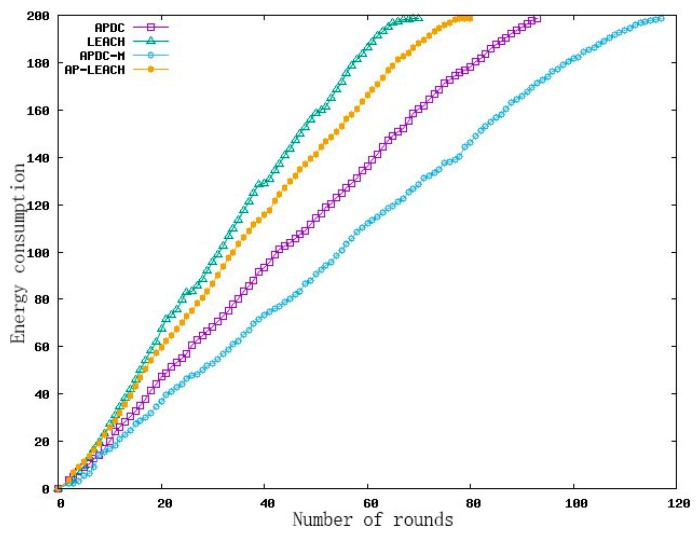
Comparison of energy consumption.

The main goal of this experiment was to compare the influence of LEACH, AP-LEACH, APDC and APDC-M on the energy consumption of the whole network. Figure 8 shows the experimental results.

The LEACH algorithm consumed all the energy in the 68th round, the AP-LEACH consumed all the energy in the 80th round, the APDC algorithm consumed all the energy in the 94th round and the APDC-M algorithm consumed all the energy in the 117th round. Compared with LEACH and AP-LEACH, APDC takes into account the position relationship between nodes to complete clustering, which can effectively reduce the energy consumption of transmission from nodes and cluster heads, and takes the residual energy of nodes as the clustering standard. The nodes with high residual energy were elected as cluster heads, which effectively balanced the energy consumption of each node in the network, thus, the effective working time of the WSN was extended. On the basis of APDC, APDC-M balances the energy consumption of each cluster head to complete data forwarding by double cluster heads and multi-hop between clusters, which reduced the energy consumption of external forwarding, and then prolonged the network life cycle as a whole.

## 6. Conclusions

In this paper, a double cluster heads and multi-hop network topology control algorithm (APDC-M) based on the AP clustering algorithm was proposed. In each cluster, two cluster heads were elected to complete data fusion and data forwarding respectively. An election strategy based on AP clustering and reference nodes was used to complete the election of fusion cluster heads, and the clustering of network nodes was completed; then the corresponding forwarding cluster head node were elected in each cluster, the forwarding cluster head node of each cluster completed the data transmission to the base station by multi-hop mode among clusters. Compared with the classical algorithm, APDC-M could make the cluster head distribution more uniform, the cluster head election more reasonable and more energy saving, and effectively reduced the energy consumption of data transmission between cluster head and base station, thus prolonging the overall network life.

## Figures and Tables

**Table 1:** Comparison of the improved algorithms about low energy adaptive clustering hierarchy (LEACH).

Algorithm	Residual Energy Consideration	Cluster Head Distribution Rationality	Energy Consumption Balance of Cluster Head	Transmission from Cluster Head to Base Station
LEACH-C	Yes	Poor	Poor	Single hop
EBCRP	No	Good	Good	Multi-hop (single head)
LEACH-EC	Yes	Poor	Poor	Single hop
O-LELACH	Yes	Poor	Poor	Single hop
LEACH-MAC	No	Poor	Poor	Single hop
IBLEACH	Yes	Poor	Good	Multi-hop (single head)
LEACH-KM-GA	Yes	Good	Poor	Single hop
EDROPL	No	Good	Good	Multi-hop (single head)
pLEACH	Yes	Good	Good	Multi-hop (single head)
AP-LEACH	No	Good	Poor	Single hop
APDC-M	Yes	Good	Good	Multi-hop (dual heads)

**Table 2:** Parameter description in formulas.

Parameter	Meaning
k	k bits of data
E_elec_	Transmission circuit energy consumption
d_i,f_	Distance from i to fusion node
d_i,bs_	Distance from i to base station
E_init_	Initial energy of a node
E_rem_	Residual energy of a node
$\varepsilon_{fs}$	Circuit energy consumption of power amplifier in free space model
$\varepsilon_{mp}$	Circuit energy consumption of power amplifier in multipath fading model

**Table 3:** Simulation experiment parameters.

Parameter	Value
E_elec_	50 nJ/bit
$\varepsilon_{fs}$	10 pJ/bit/m^2^
$\varepsilon_{mp}$	0.0013 pJ/bit/m^4^
Data fusion rate	0.5
Number of networks nodes	100
Size of data packet	4016 bit
E_init_	2 J
Monitoring area	100 m × 100 m

**Table 4:** Parameter description of energy consumption model.

Parameter	Meaning
k	k bits of data
E_elec_	Transmission circuit energy consumption
d	Distance between transmitting node and receiving node
d_0_	Distance threshold
$\varepsilon_{fs}$	Circuit energy consumption of power amplifier in free space model
$\varepsilon_{mp}$	Circuit energy consumption of power amplifier in multipath fading model

**Table 5:** Comparison of clustering data.

Algorithm	Number of Cluster Heads	Average Distance Between a Node and Cluster Head/m	Maximum Number of Nodes in Cluster	Minimum Number of Nodes in Cluster
LEACH	6	22.47	21	7
APDC	8	12.34	16	10

## Abbreviations

The following abbreviations are used in this manuscript:

HEED	Hybrid, Energy-Efficient, Distributed clustering approach
LEACH	Low Energy Adaptive Clustering Hierarchy
AP	Affinity Propagation clustering
LEACH-C	Low Energy reconciling agglomeration Hierarchy-Centralized
EBCRP	Energy Balanced Clustering Routing Protocol
LEACH-EC	Low Energy reconciling agglomeration Hierarchy-Energy and Connectivity
O-LEACH	Optimized Low Energy reconciling agglomeration Hierarchy
LEACH-MAC	LEACH-Media Access Control
IBLEACH	Intra-Balanced Low Energy reconciling agglomeration Hierarchy
LEACH-KM-GA	LEACH based on K-Means and Gauss Algorithms
EDROPL	Routing algorithm based on Energy Consumption Gradient
pLEACH	partition-based Low Energy reconciling agglomeration Hierarchy
AP-LEACH	Low Energy reconciling agglomeration Hierarchy based on Affinity Propagation
APDC	Double Choices algorithm based on Affinity Propagation and reference node
APDC-M	Double Cluster heads and Multi-hop based on Affinity Propagation
SPFA	Shortest Path Faster Algorithm
